# Loss of angiopoietin-like 4 (ANGPTL4) in mice with diet-induced obesity uncouples visceral obesity from glucose intolerance partly via the gut microbiota

**DOI:** 10.1007/s00125-018-4583-5

**Published:** 2018-03-03

**Authors:** Aafke W. F. Janssen, Saeed Katiraei, Barbara Bartosinska, Daniel Eberhard, Ko Willems van Dijk, Sander Kersten

**Affiliations:** 1 Nutrition, Metabolism and Genomics Group, Division of Human Nutrition and Health, Wageningen University, Stippeneng 4, 6708 WE Wageningen, the Netherlands; 2 Department of Human Genetics, Leiden University Medical Center, Leiden, the Netherlands; 3 Einthoven Laboratory for Experimental Vascular Medicine, Leiden University Medical Center, Leiden, the Netherlands; 4 Institute of Metabolic Physiology, Department of Biology, Heinrich Heine University Düsseldorf, Düsseldorf, Germany; 5 Institute for Beta Cell Biology, German Diabetes Center, Leibniz Center for Diabetes Research, Heinrich Heine University Düsseldorf, Düsseldorf, Germany; 6 German Center for Diabetes Research (DZD), München Neuherberg, Germany; 7 Division of Endocrinology, Department of Medicine, Leiden University Medical Center, Leiden, the Netherlands

**Keywords:** Angiopoietin-like 4, Antibiotics, Glucose tolerance, Gut microbiota, Insulin secretion, White adipose tissue

## Abstract

**Aims/hypothesis:**

Angiopoietin-like 4 (ANGPTL4) is an important regulator of triacylglycerol metabolism, carrying out this role by inhibiting the enzymes lipoprotein lipase and pancreatic lipase. ANGPTL4 is a potential target for ameliorating cardiometabolic diseases. Although ANGPTL4 has been implicated in obesity, the study of the direct role of ANGPTL4 in diet-induced obesity and related metabolic dysfunction is hampered by the massive acute-phase response and development of lethal chylous ascites and peritonitis in *Angptl4*^−/−^ mice fed a standard high-fat diet. The aim of this study was to better characterise the role of ANGPTL4 in glucose homeostasis and metabolic dysfunction during obesity.

**Methods:**

We chronically fed wild-type (WT) and *Angptl4*^−/−^ mice a diet rich in unsaturated fatty acids and cholesterol, combined with fructose in drinking water, and studied metabolic function. The role of the gut microbiota was investigated by orally administering a mixture of antibiotics (ampicillin, neomycin, metronidazole). Glucose homeostasis was assessed via i.p. glucose and insulin tolerance tests.

**Results:**

Mice lacking ANGPTL4 displayed an increase in body weight gain, visceral adipose tissue mass, visceral adipose tissue lipoprotein lipase activity and visceral adipose tissue inflammation compared with WT mice. However, they also unexpectedly had markedly improved glucose tolerance, which was accompanied by elevated insulin levels. Loss of ANGPTL4 did not affect glucose-stimulated insulin secretion in isolated pancreatic islets. Since the gut microbiota have been suggested to influence insulin secretion, and because ANGPTL4 has been proposed to link the gut microbiota to host metabolism, we hypothesised a potential role of the gut microbiota. Gut microbiota composition was significantly different between *Angptl4*^−/−^ mice and WT mice. Interestingly, suppression of the gut microbiota using antibiotics largely abolished the differences in glucose tolerance and insulin levels between WT and *Angptl4*^−/−^ mice.

**Conclusions/interpretation:**

Despite increasing visceral fat mass, inactivation of ANGPTL4 improves glucose tolerance, at least partly via a gut microbiota-dependent mechanism.

**Electronic supplementary material:**

The online version of this article (10.1007/s00125-018-4583-5) contains peer-reviewed but unedited supplementary material, which is available to authorised users.

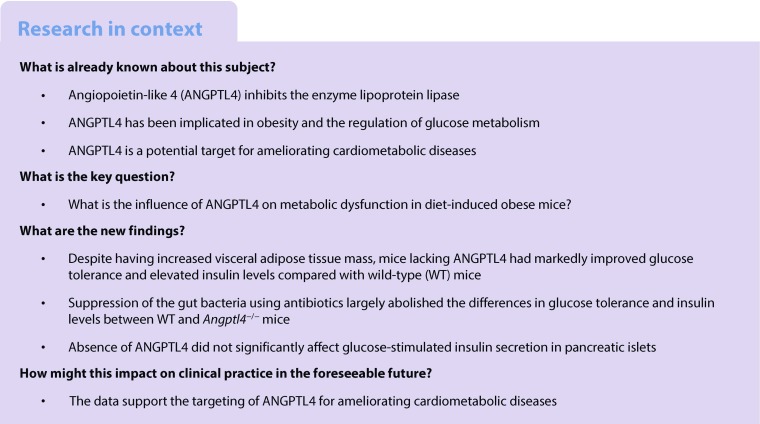



## Introduction

Obesity is often accompanied by insulin resistance, which greatly increases the risk of cardiometabolic complications [1]. Part of the effect of insulin resistance on cardiometabolic risk is conferred by changes in plasma lipoproteins. A particular class of lipoproteins are triacylglycerol-rich lipoproteins, consisting of chylomicrons and VLDL [2]. Once in the blood, triacylglycerol-rich lipoproteins are quickly captured and hydrolysed by lipoprotein lipase (LPL) in the capillaries of fat and muscle tissue [3–5]. The resulting fatty acids are taken up by underlying fat and muscle cells, and are either stored as triacylglycerols or used as fuel [3]. To ensure that the rate of uptake of fatty acids matches local energy demands, the activity of LPL is carefully regulated. An important group of physiological regulators of LPL activity are the angiopoietin-like proteins, including the ubiquitously expressed angiopoietin-like 4 (ANGPTL4) [6]. We and others have shown that ANGPTL4 post-translationally inhibits LPL activity under various physiological conditions, including fasting and cold exposure, thereby raising plasma triacylglycerol levels [7–9].

Besides inhibiting LPL, ANGPTL4 also inhibits pancreatic lipase, the main enzyme responsible for hydrolysis of dietary triacylglycerols in the gastrointestinal tract and a structural homologue of LPL. Consequently, mice lacking ANGPTL4 have enhanced dietary lipid absorption compared with wild-type (WT) mice, contributing to the higher fat mass and body weight [10].

By reducing fat storage in adipose tissue, ANGPTL4 might promote ectopic fat storage in non-adipose tissues, which in turn could impact on glucose homeostasis. Indeed, a number of mouse studies have implicated ANGPTL4 in the regulation of glucose metabolism. While *Angptl4* inactivation did not influence plasma glucose and insulin levels [11], liver-specific overexpression of *Angptl4* by adenovirus lowered circulating glucose levels and improved glucose tolerance, possibly via reduced hepatic glucose production [12, 13]. In contrast, whole-body transgenic overexpression of *Angptl4* decreased glucose tolerance after prolonged high-fat feeding [14]. In clamp studies, whole-body transgenic *Angptl4* overexpression led to impaired glucose utilisation and insulin resistance in the periphery, and higher insulin-mediated suppression of glucose production in the liver [15]. Hence, published data do not present a uniform picture on the influence of ANGPTL4 on glucose metabolism and insulin sensitivity.

Considering the involvement of ANGPTL4 in fat uptake and storage, and its potential role in glucose metabolism, it is of interest to investigate the role of ANGPTL4 in metabolic dysfunction and insulin sensitivity during obesity. Unfortunately, the study of ANGPTL4 in diet-induced obesity and associated metabolic dysfunction is hampered by the massive acute phase response and the development of lethal chylous ascites in *Angptl4*^−/−^ mice fed a high-fat diet. This severe inflammatory response is specifically provoked by a diet high in saturated fatty acids [16]. By contrast, feeding *Angptl4*^−/−^ mice a diet rich in *cis* or *trans* unsaturated fatty acids does not trigger an inflammatory response [17]. Accordingly, in the present paper we investigated the influence of ANGPTL4 on diet-induced obesity and metabolic dysfunction by feeding WT and *Angptl4*^−/−^ mice a diet rich in unsaturated fatty acids, followed by detailed investigation of the metabolic phenotype. Cholesterol was added to the diet and fructose to the drinking water to mimic a western-style diet.

## Methods

### Animals and diet

Animal studies were performed using purebred WT and *Angptl4*^−/−^ mice on a C57Bl/6 background that were bred and maintained in the same facility for more than 20 generations [11]. *Angptl4*^−/−^ mice were generated via homologous recombination of embryonic stem cells, and lack part of the *Angptl4* gene, resulting in a non-functional ANGPTL4 protein [11]. The *Angptl4*^−/−^ mice were imported to our animal facility in 2006 as strain B6.129P2-Lp139tm1 N10 from Taconic (Germantown, NY, USA; a kind gift of A. Köster, Eli Lilly, Indianapolis, IN, USA). Mice were individually housed in temperature- and humidity-controlled specific pathogen-free conditions. Mice had ad libitum access to food and water.

In study 1, two groups of mice were studied; male WT and *Angptl4*^−/−^ mice at 10–13 weeks of age. These two groups were part of a larger study (the control groups of this study), which aimed to investigate the effects of different dietary fibres on non-alcoholic fatty liver disease in WT and *Angptl4*^−/−^ mice [18]. The assignment to the different fibre groups was randomised using an online randomisation tool. Liver fat levels, liver histology and hepatic expression of genes related to inflammation (e.g. *F4/80* [also known as *Adgre1*], *Cd68*, *Mcp-1* [*Cxcl2*], *Il1b*, *Il1ra* [*Il1rn*], *Tnfa* [*Tnf*], *Itgax*, *Timp1*) were all analysed (all were significantly higher in *Angptl4*^−/−^ mice compared with WT mice; data not included). A power calculation was performed based on plasma alanine aminotransferase (ALT) activity as a biomarker for liver health. Assuming a plasma ALT activity of 40 U/l in WT mice with a *σ* value of 16, using a power of 0.8, a significance level of 0.05, and an effect size of 20 U/l, the sample size was calculated as *n* = 11 mice per group. To allow compensation for potential loss of mice during the study, *n* = 12 mice were included per group. Mice were fed a high-fat/high-cholesterol/high-fructose diet, providing 45% energy as triacylglycerols (Formula D12451, Research Diets; manufactured by Research Diet Services, Wijk bij Duurstede, the Netherlands; sterilised with γ-irradiation at 9 kGy) for 18 weeks [18–20]. The fat source of the diet was replaced by safflower oil and supplemented with 1% cholesterol (wt/wt) (Dishman, Veenendaal, the Netherlands). Fructose was administered by adding 20% fructose (wt/vol.) to the drinking water. After 17 weeks of dietary intervention, all mice underwent a glucose tolerance test, as explained below.

In study 2, male WT and *Angptl4*^−/−^ mice at 19–22 weeks of age received the same diet as in study 1 (Formula 58V8 a.k.a. D12541; manufactured by TestDiet, Saint Louis, MO, USA; sterilised with γ-irradiation at 18–50 kGy) for 18 weeks. The fat source of the diet was replaced by safflower oil and supplemented with 1% cholesterol (wt/wt). Fructose was administered by adding 20% fructose (wt/vol.) to the drinking water. A mixture of broad-spectrum antibiotics was provided in the drinking water (1 g/l ampicillin, 1 g/l neomycin sulfate and 0.5 g/l metronidazole). This antibiotic cocktail was previously shown to effectively suppress intestinal bacteria [21, 22]. Control mice received normal water. The assignment of the antibiotics/normal water was randomised using an online randomisation tool. After 15 weeks of dietary intervention, all mice underwent an intestinal permeability assay (see below for further details). After 16 weeks, all mice underwent an insulin tolerance test, and after 17 weeks a glucose tolerance test, as explained below. A power calculation was performed based on the AUC of the glucose tolerance test; assuming an AUC of 1800 mmol/l × min in WT mice with a *σ* value of 150, using a power of 0.8, a significance level of 0.05, and an effect size of 200 mmol/l × min, the sample size was calculated as *n* = 9 mice per group. To allow compensation for potential loss of mice during the study, *n* = 10 mice were included per group. PCR indicated that two mice in the WT group treated with antibiotics were in fact *Angptl4*^−/−^ mice. These two mice were included in the group of *Angptl4*^−/−^ mice on antibiotics.

Body weight and food intake were assessed weekly in both studies. After 18 weeks, mice were anaesthetised using isoflurane and blood was collected by orbital puncture. Mice were euthanised via cervical dislocation, after which tissues were excised and weighed and intestinal content was sampled. Samples were immediately frozen in liquid nitrogen and stored at −80°C. All animal experiments were approved by the local animal ethics committee of Wageningen University, the Netherlands. All analyses were conducted with the experimenter blinded to group assignment.

### Intraperitoneal glucose tolerance test

In both study 1 and 2, a glucose tolerance test was performed 1 week before mice were euthanised. The test was carried out as previously described [14]. Briefly, after 5 h of fasting, mice were injected i.p. with glucose (0.8 g/kg body weight) and blood glucose and insulin levels were measured at various time points during the test (see electronic supplementary materials (ESM) Methods for further details).

### Insulin tolerance test

In study 2, an insulin tolerance test was performed 2 weeks before mice were euthanised. The test was carried out as previously described [14]. Briefly, blood samples were collected immediately before and at selected time points after i.p. insulin injection (0.75 U/kg body weight) (see ESM Methods for further details).

### Intestinal permeability assay

In study 2, an intestinal permeability assay was performed 3 weeks before euthanasia. FITC-dextran (4 kD) (Sigma, Houten, the Netherlands) was administered by oral gavage (600 mg/kg body weight, 40 mg/ml). After 1 h, blood was drawn, stored on ice in the dark and centrifuged for 15 min at 1000 *g*. The plasma obtained was diluted in PBS and fluorescence intensity was measured using a fluorescence spectrophotometer (Fluoroskan Ascent; Thermo Fisher Scientific, Breda, the Netherlands; excitation wavelength (*λ*_ex_), 485 nm; emission wavelength (*λ*_em_), 538 nm). FITC-dextran concentrations were determined using a standard curve generated by diluting FITC-dextran in PBS. A baseline blood sample was used to correct for autofluorescence.

### Plasma measurements

Serum amyloid A was measured via ELISA (Life technologies, Bleiswijk, the Netherlands), plasma triacylglycerol by a commercially available colourimetric kit (HUMAN Diagnostics, Wiesbaden, Germany) and endotoxin levels via the Limulus Amebocyte Lysate assay (Lonza, Walkerville, MD, USA).

### Cell culture

Mouse beta-TC6 cells (CRL11506; ATCC via LGC [Wesel, Germany]) were grown in 24-well plates in Dulbecco’s modified Eagle’s medium (DMEM) supplemented with 15% heat-inactivated FCS and 1% penicillin/streptomycin (Lonza, Verviers, Belgium) under 5% CO_2_ at 37°C. Cells tested negative for mycoplasma.

### RNA isolation and PCR

Total RNA was extracted from mesenteric adipose tissue, beta-TC6 cells and mouse pancreatic islets using TRIzol reagent (Life technologies) and the RNeasy minikit (Qiagen, Venlo, The Netherlands). RNA was reverse-transcribed using the iScript cDNA synthesis kit (Bio-Rad Laboratories, Veenendaal, the Netherlands). PCR was carried out as previously described [7] for the following genes: *36b4* (also known as *Rplp0*), *Cd68*, *F4/80*, *Mcp-1*, *Il6*, *Il1ra*, *Lpl* and *Angptl4*. Further details can be found in ESM Methods.

### Mouse pancreatic islet isolation

Pancreatic islets were isolated from WT and *Angptl4*^−/−^ mice by injecting Liberase TL (Sigma) into the bile duct. Additional details can be found in ESM Methods.

### Glucose-stimulated insulin secretion

Groups of 8 islets isolated from WT and *Angptl4*^−/−^ mice were fasted for 1 h, followed by stimulation with low (2 mmol/l) and high (20 mmol/l) glucose. Islets were lysed, and secreted insulin and insulin content was quantified by ELISA (Crystal Chem, Zaandam, the Netherlands) (see ESM Methods).

### ^1^H-NMR spectroscopy

Short-chain fatty acid (SCFA) levels in the content of the caecum were measured using an Avance III NMR spectrometer (Bruker, Leiderdorp, the Netherlands), as previously described [18].

### Immunohistochemistry

Paraffin-embedded pancreas sections (5 μm) were immunohistochemically stained using anti-insulin antibody (Insulin (H-86): sc-9168; Santa Cruz Biotechnology, Dallas, TX, USA; 1:800), followed by a biotinylated goat anti-rabbit antibody (BA-1000; Vector Labs, Brunschwig Chemie, Amsterdam, the Netherlands; 1:200), and an avidin-biotin-complex (ABC) coupled to peroxidase (Vector Labs). Visualisation was carried out using 3,3′-diaminobenzidine for 5 min. For negative controls, primary antibodies were omitted.

### LPL activity measurements

LPL activity in mesenteric white adipose tissue was quantified using a [^3^H]oleic acid-labelled triolein substrate, as previously described [9].

### Western blot

Western blotting was performed to detect LPL. Mesenteric fat pads were lysed in ice-cold Pierce IP lysis buffer (Thermo Fisher Scientific) containing protease and phosphatase inhibitors (Roche, Almere, the Netherlands). Lysates were centrifuged to remove fat droplets before protein separation and immunoblotting with goat anti-mouse LPL or rabbit anti-goat HSP90 (Cell Signaling, Danvers, MA, USA), both at 1:2000. Further processing of samples was conducted as outlined previously [7] and described in ESM Methods.

### DNA extraction

Faecal samples were resuspended in buffer and cells were lysed. DNA was subsequently extracted using either phenol:chloroform:isoamyl alcohol [25:24:1] or the Maxwell 16 System (Promega, Madison, WI, USA) (see ESM Methods).

### *16S* rRNA gene sequencing

For *16S* ribosomal RNA (rRNA) gene sequencing, DNA samples were sent to the Broad Institute of MIT and Harvard (Cambridge, MA, USA) and processed as previously described [18]. For statistical significance, biological relevance and visualisation, the linear discriminant analysis (LDA) effect size (LEfSe) method was used. The sequencing data are available from the authors upon request. Further details are provided in ESM Methods.

### Bacterial *16S* rRNA gene quantification

Real-time PCR was performed on faecal DNA samples from study 2 to investigate *16S* rRNA gene expression, using amplified and purified *16S* rRNA to generate a standard curve (see ESM Methods for details).

### Statistics

Data are presented as mean ± SEM, unless otherwise indicated. Statistical analyses were performed using an unpaired Student’s *t* test or two-way ANOVA followed by a Bonferroni test for post-hoc analysis (GraphPad Software, La Jolla, CA, USA). A *p* value <0.05 was considered statistically significant.

## Results

**Fig. 1:**
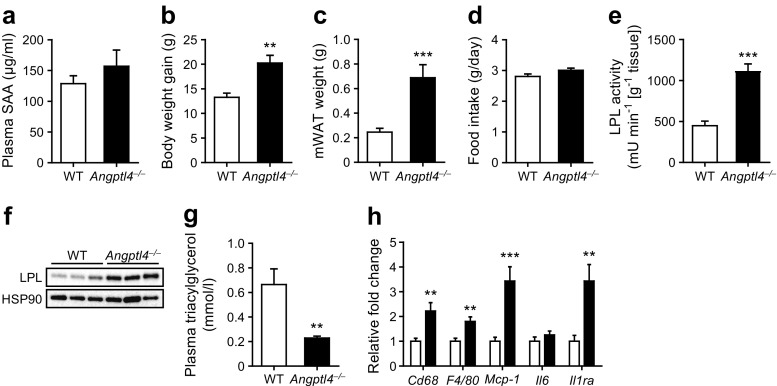
Loss of ANGPTL4 promotes diet-induced obesity and adipose tissue inflammation. (**a**) Plasma serum amyloid A (SAA) concentration. (**b**) Body weight gain of WT and *Angptl4*^−/−^ mice at the end of the dietary intervention. (**c**) Mesenteric white adipose tissue (mWAT) weight. (**d**) Average food intake per mouse per day during the dietary intervention. (**e**) LPL activity and (**f**) LPL protein levels in mWAT homogenates. (**g**) Plasma triacylglycerol levels. (**h**) Relative expression of inflammatory genes in mWAT of WT (white bars) and *Angptl4*^−/−^ mice (black bars). Gene expression levels of WT mice were set at 1. Data are presented as mean ± SEM. *n* = 12 mice per group. **p < 0.01, ***p < 0.001, Student’s *t* test

To elicit a phenotype that includes (visceral) obesity, insulin resistance and non-alcoholic fatty liver disease, in study 1 WT and *Angptl4*^−/−^ mice were fed a diet rich in unsaturated fatty acids and cholesterol, complemented with fructose in the drinking water [18–20]. Plasma serum amyloid A concentrations were low in *Angptl4*^−/−^ mice fed a diet rich in unsaturated fatty acids compared with *Angptl4*^−/−^ mice fed a diet rich in saturated fatty acids [16, 17], and were not significantly elevated compared with WT mice (Fig. 1a). *Angptl4*^−/−^ mice gained more weight and had a higher visceral (mesenteric) fat mass compared with WT mice, despite a similar level of food intake (Fig. 1b–d). As expected, LPL activity and the amount of LPL protein were significantly higher in mesenteric fat of *Angptl4*^−/−^ mice compared with WT mice (Fig. 1e, f). In addition, plasma triacylglycerol levels were significantly lower in *Angptl4*^−/−^ mice than in WT mice (Fig. 1g). Consistent with the increased body weight and elevated mesenteric fat-pad weight, *Angptl4*^−/−^ mice displayed increased inflammation in mesenteric fat compared with WT mice, as revealed by higher expression of several inflammatory genes (Fig. 1h).

### Loss of ANGPTL4 leads to improved glucose tolerance

**Fig. 2:**
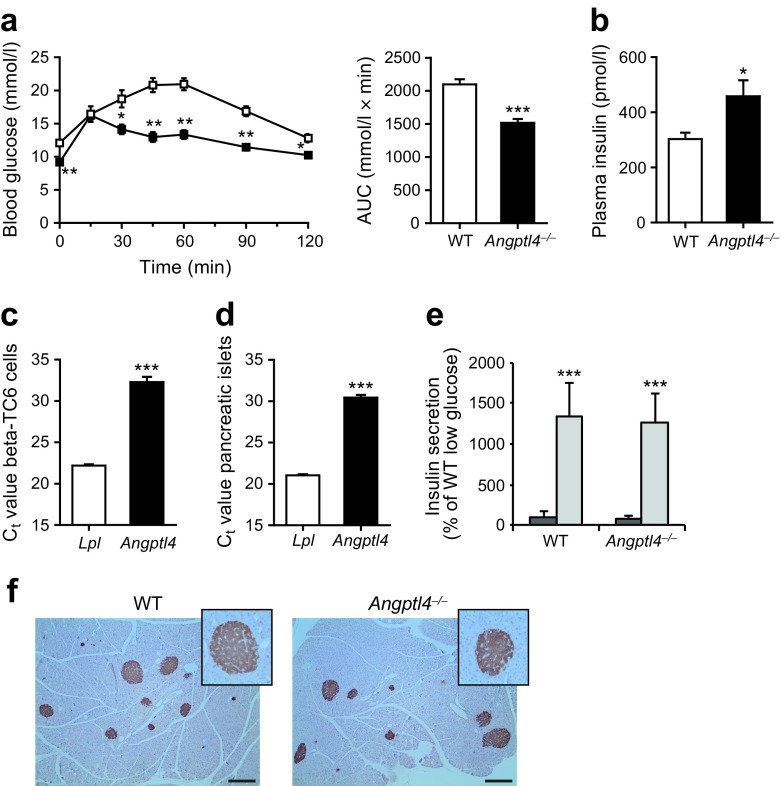
Loss of ANGPTL4 leads to improved glucose tolerance. (**a**) i.p. glucose tolerance test and AUC of WT (white squares) and *Angptl4*^−/−^ (black squares) mice 1 week before euthanasia. (**b**) Fasting plasma insulin levels. (**c**–**d**) C_t_ values of *Lpl* and *Angptl4* in (**c**) mouse beta-TC6 cells and (**d**) pancreatic islets isolated from C57Bl/6 mice. (**e**) Glucose-stimulated insulin secretion in pancreatic islets isolated from WT and *Angptl4*^−/−^ mice. Secreted insulin measured in supernatants was normalised to the protein content in the cell lysate and expressed as percentage of the mean of levels in WT islets treated with 2 mmol/l glucose. Dark grey bars, low glucose stimulation (2 mmol/l); light grey bars, high glucose stimulation (20 mmol/l). Number of biological replicates: WT low glucose, *n* = 12; *Angptl4*^−/−^ low glucose, *n* = 11; WT high glucose, *n* = 12; *Angptl4*^−/−^ low glucose, *n* = 10. (**f**) Representative pictures of pancreas stained for insulin (brown staining). Scale bar, 200 μm. Insets show a higher magnification of a pancreatic islet. Data are presented as mean ± SEM (*n* = 12 mice per group) except for the data from beta-TC6 cells and pancreatic islets (SD). *p < 0.05, **p < 0.01, ***p < 0.001, Student’s *t* test

Strikingly, despite the higher visceral fat mass and inflammation, blood glucose levels during the glucose tolerance test were markedly lower in *Angptl4*^−/−^ mice than in WT mice (Fig. 2a). The lower glucose levels in *Angptl4*^−/−^ mice were accompanied by elevated fasting plasma insulin levels (Fig. 2b). These data suggest that *Angptl4*^−/−^ mice are more glucose tolerant than WT mice, possibly owing to higher plasma insulin levels.

To explore the potential origin of the elevated plasma insulin levels in *Angptl4*^−/−^ mice, we investigated whether ANGPTL4 might be expressed in pancreatic islets, together with LPL, thereby influencing insulin secretion. LPL has been proposed as a mediator of the effect of dietary lipids on insulin secretion [23]. However, although *Lpl* expression was relatively high in primary pancreatic islets and in the beta cell line beta-TC6, with C_t_ values of ~21–22, *Angptl4* expression was very low, with C_t_ values of ~30.5–32 (Fig. 2c, d). Hence, the effect of ANGPTL4 on glucose tolerance is unlikely to be mediated by ANGPTL4 produced by beta cells. BioGPS supports the minimal expression of *Angptl4* in the mouse pancreas (www.biogps.org/, accessed 11 May 2017). Furthermore, analysis of several microarray datasets (www.ncbi.nlm.nih.gov/gds) of mouse pancreatic islets (GSE43620), the beta cell line beta-TC3 (GSE31102) and beta cells from adult mice (GSE54374) confirmed that *Angptl4* is expressed at very low levels in pancreatic islets and beta cells. Consistent with the minimal expression of *Angptl4* in pancreatic islets, glucose-stimulated insulin secretion was not significantly altered in isolated pancreatic islets of *Angptl4*^−/−^ mice compared with WT mice (Fig. 2e). In addition, insulin staining of the pancreas showed no difference in the abundance of pancreatic islets or insulin staining intensity in WT vs *Angptl4*^−/−^ mice (Fig. 2f).

Notably, the above data do not exclude a role for circulating ANGPTL4 in the regulation of islet LPL activity, and in insulin secretion. However, upregulation of LPL activity in beta cells has been shown to lead to hyperglycaemia during a glucose tolerance test [24]. Accordingly, it is unlikely that the observed hypoglycaemia and hyperinsulinaemia in *Angptl4*^−/−^ mice are linked to loss of inhibition of LPL activity in islet cells, whether via ANGPTL4 that is produced in the pancreas or elsewhere. Together, these findings suggest that ANGPTL4 probably does not influence plasma insulin levels via a direct effect on insulin secretion in pancreatic islets.

### Loss of ANGPTL4 alters levels of gut-derived metabolites and changes gut microbial composition

**Fig. 3:**
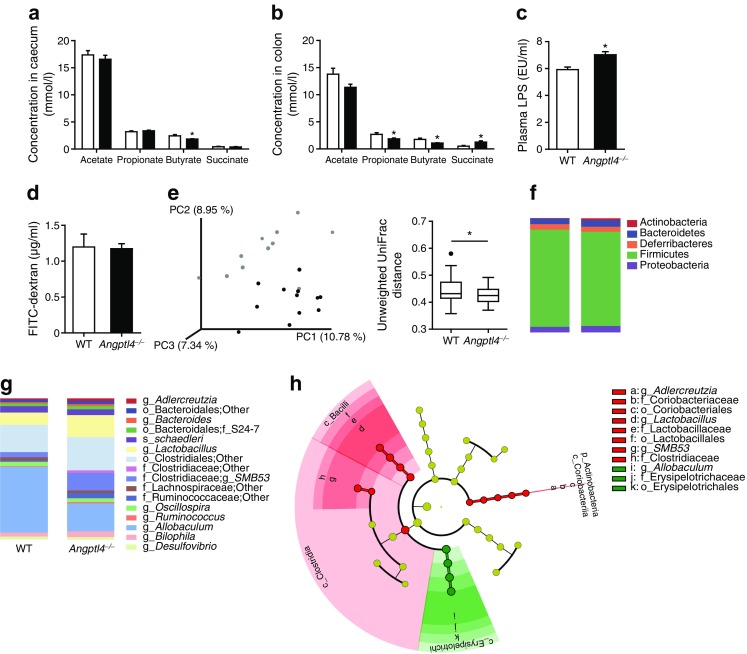
Loss of ANGPTL4 alters levels of gut-derived metabolites and changes gut microbial composition. (**a**–**b**) Concentration of acetate, propionate, butyrate and succinate in (**a**) the caecum and (**b**) the colon of WT (white bars) and *Angptl4*^−/−^ (black bars) mice. *n* = 12 mice per group. (**c**) LPS levels (endotoxin units [EU]/ml) in plasma of WT and *Angptl4*^−/−^ mice. *n* = 12 mice per group. (**d**) Intestinal permeability as assessed by plasma FITC-dextran concentration 1 h after oral administration. *n* = 9 mice per group. Data in (**a**–**d**) are presented as mean ± SEM. (**e**) Principal coordinates analysis plot (grey dots, WT; black dots *Angptl4*^−/−^) and box plots of unweighted UniFrac distances of the intestinal microbiota of WT (*n* = 11) and *Angptl4*^−/−^ (*n* = 12) mice. The box extends from the 25th to 75th percentiles, the line indicates the median, and a Bonferroni test was used to plot the whiskers and the outlier (solid black dot). (**f**–**g**) Mean relative abundance of the colonic bacteria (**f**) at phylum level and (**g**) at the lowest identifiable level. (**h**) Cladogram generated using the linear discriminant analysis (LDA) effect size (LEfSe) method displaying bacterial taxa significantly enriched in WT (green) or *Angptl4*^−/−^ (red) mice. The domain bacteria is depicted by the central point and each ring represents the next lower taxonomic level (phylum to genus). In (**g**) and (**h**), the letter preceding the underscore indicates the taxonomic level: c, class; p, phylum; o, order; f, family; g, genus; s, species. *p < 0.05, Student’s *t* test

Considering that the gut microbiota may influence insulin secretion [25, 26], and since ANGPTL4 has been linked to the gut microbiota [27, 28], we hypothesised a potential role of the gut microbiota in the effect of loss of ANGPTL4 on insulin levels. To explore the possibility that *Angptl4*^−/−^ mice have a different gut microbiota to WT mice, in study 1 we first measured the levels of several gut-derived metabolites, including luminal succinate and SCFAs, and plasma lipopolysaccharide (LPS). In the caecum, the concentration of butyrate was significantly lower in *Angptl4*^−/−^ mice than in WT mice (Fig. 3a). Moreover, in the colon, levels of both propionate and butyrate were significantly lower, and levels of succinate were significantly higher in *Angptl4*^−/−^ mice (Fig. 3b). Also, plasma LPS levels were higher in *Angptl4*^−/−^ mice than in WT mice, even though intestinal permeability measured using FITC-dextran was similar (Fig. 3c, d). Together, these findings suggest a potential difference in gut microbiota composition between *Angptl4*^−/−^ and WT mice.

To further examine the gut microbiota in WT and *Angptl4*^−/−^ mice in study 1, we performed *16S* rRNA sequencing. Principal coordinate analysis of unweighted UniFrac distance demonstrated differential clustering of microbiome sequences in *Angptl4*^−/−^ and WT mice (Fig. 3e). The majority of the difference in bacterial community between *Angptl4*^−/−^ and WT mice can be explained by the phyla Actinobacteria and Firmicutes. Indeed, only small non-significant differences were observed within the phyla Bacteroidetes, Deferribacteres and Proteobacteria. The phylum Actinobacteria was 2.5-fold more abundant in *Angptl4*^−/−^ mice, which was mainly accounted for by a significant increase in the genus *Adlercreutzia.* While the relative abundance of the phylum Firmicutes was comparable between the genotypes, the genera *Lactobacillus* and *SMB53* were over-represented in *Angptl4*^−/−^ mice. In contrast, consistent with the lower colonic butyrate levels, the butyrate-producing *Allobaculum* was less abundant in *Angptl4*^−/−^ mice (Fig. 3f–h, ESM Table 1), whereas the abundance of other butyrate-producing bacteria was not different (members of Lachnospiraceae) or was increased (members of Clostridiaceae family). Overall, these data indicate that the gut bacterial composition is significantly different between *Angptl4*^−/−^ and WT mice, which may explain the different levels of gut-derived metabolites.

### Suppression of gut bacteria abolishes the increase in glucose tolerance in Angptl4^−/−^ mice

**Fig. 4:**
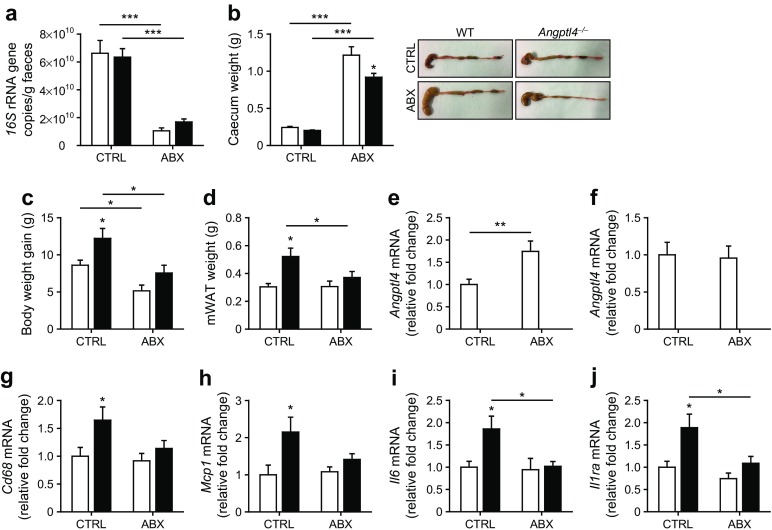
Antibiotics largely abolish the difference in adipose tissue inflammation between WT and *Angptl4*^−/−^ mice. Mice were fed a high-fat/high-cholesterol/high-fructose diet either without (CTRL) or with (ABX) antibiotic supplementation. (**a**) *16S* rRNA gene copies per g of faeces in WT and *Angptl4*^−/−^ mice with or without antibiotic supplementation. WT mice, white bars; *Angptl4*^−/−^ mice, black bars. (**b**) Weight and representative images of the caecum. (**c**) Body weight gain at the end of the dietary intervention with or without antibiotic supplementation. (**d**) Weight of mesenteric white adipose tissue (mWAT). (**e**–**f**) Relative mRNA levels of *Angptl4* in (**e**) the ileum or (**f**) mWAT. (**g**–**j**) Relative expression of the inflammatory genes (**g**) *Cd68*, (**h**) *Mcp1*, (**i**) *Il6* and (**j**) *Il1ra* in mWAT. Gene expression levels of WT mice fed the control diet without antibiotic supplementation were set at 1. Data are presented as mean ± SEM. WT ABX, *n* = 8; WT CTRL, *n* = 10; *Angptl4*^−/−^ CTRL, *n* = 10; *Angptl4*^−/−^ ABX, *n* = 12. *p < 0.05, **p < 0.01, ***p < 0.001 vs WT or as indicated, two-way ANOVA with Bonferroni post-hoc test

To further study the impact of the gut microbiota on the effect of *Angptl4* ablation on glucose tolerance, we repeated the diet-induced obesity study in WT and *Angptl4*^−/−^ mice with or without antibiotics in their drinking water (study 2). Antibiotic supplementation significantly reduced the number of *16S* rRNA gene copies in the faeces (Fig. 4a) and markedly increased caecum weight (Fig. 4b), indicating an effective suppression of gut bacteria.

Overall body weight gain was higher in *Angptl4*^−/−^ mice than WT mice, which reached significance in the absence of antibiotics (Fig. 4c). In agreement with the first study, in the absence of antibiotics, *Angptl4*^−/−^ mice had elevated mesenteric fat-pad weight. In contrast, after antibiotic treatment, the mesenteric fat mass was not significantly different between *Angptl4*^−/−^ mice and WT mice (Fig. 4d). Interestingly, in WT mice, antibiotic treatment increased *Angptl4* mRNA levels in the ileum but not in mesenteric fat tissue, suggesting intestinal *Angptl4* expression is suppressed by the gut bacteria (Fig. 4e, f).

Concurrent with the elevated fat mass, in the absence of antibiotics the expression of several inflammatory genes, including *Cd68, Mcp1, Il6* and *Il1ra*, was elevated in mesenteric fat of *Angptl4*^−/−^ mice compared with WT mice. However, in the presence of antibiotics the difference in adipose tissue inflammation between the genotypes was largely abolished (Fig. 4g–j), suggesting that the increased fat mass and associated elevation of adipose tissue inflammation in *Angptl4*^−/−^ mice are partly dependent on the gut bacteria.

**Fig. 5:**
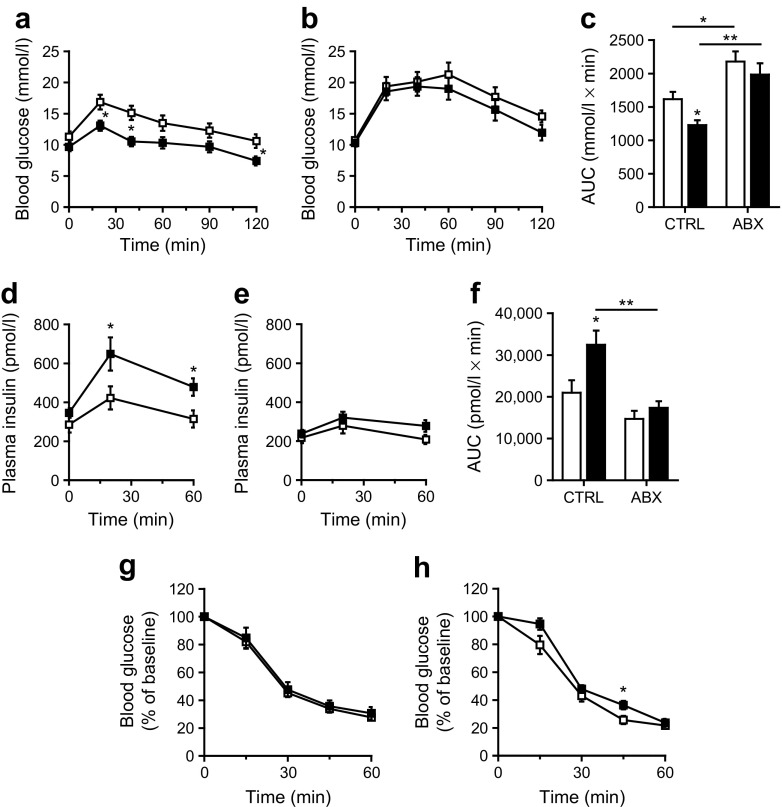
Antibiotics largely abolish the difference in glucose tolerance between WT and *Angptl4*^−/−^ mice. Mice were fed a high-fat/high-cholesterol/high-fructose diet either without (CTRL) or with (ABX) antibiotic supplementation. (**a**–**c**) i.p. glucose tolerance tests in WT (white squares/bars) and *Angptl4*^−/−^ (black squares/bars) mice fed the diet (**a**) without or (**b**) with antibiotic supplementation. (**c**) AUC of plasma glucose during the glucose tolerance tests. (**d**–**f**) Plasma insulin levels during the glucose tolerance test in WT (white squares/bars) and *Angptl4*^−/−^ (black squares/bars) mice fed the diet, (**d**) without or (**e**) with antibiotic supplementation. (**f**) AUC of plasma insulin during the glucose tolerance test. (**g**–**h**) Blood glucose levels as a percentage of baseline during an insulin tolerance test in WT (white squares) and *Angptl4*^−/−^ (black squares) mice fed the diet (**g**) without or (**h**) with antibiotic supplementation. Data are presented as mean ± SEM. WT ABX, *n* = 8; WT CTRL, *n* = 10; *Angptl4*^−/−^ CTRL, *n* = 10; *Angptl4*^−/−^ ABX, *n* = 12. *p < 0.05 **p < 0.01 vs WT or as indicated, Student’s *t* test or two-way ANOVA with Bonferroni post-hoc test

Consistent with data presented in Fig. 2a, in study 2 blood glucose levels during the glucose tolerance test were significantly lower in *Angptl4*^−/−^ mice than in WT mice (Fig. 5a). Suppression of the gut bacteria using antibiotics substantially reduced the differences in blood glucose levels between the two sets of mice, suggesting a role for the gut microbiota in the effect of ANGPTL4 on glucose tolerance (Fig. 5b, c). The lower glucose levels in *Angptl4*^−/−^ mice were accompanied by higher plasma insulin levels (Fig. 5d). Insulin tolerance was not different between *Angptl4*^−/−^ and WT mice (Fig. 5e, f), irrespective of antibiotic treatment (Fig. 5g, h). Overall, our findings suggest that loss of ANGPTL4 promotes (visceral) obesity yet, by raising insulin levels, reduces glucose intolerance, and that this effect is partly dependent on the gut bacteria.

## Discussion

**Fig. 6:**
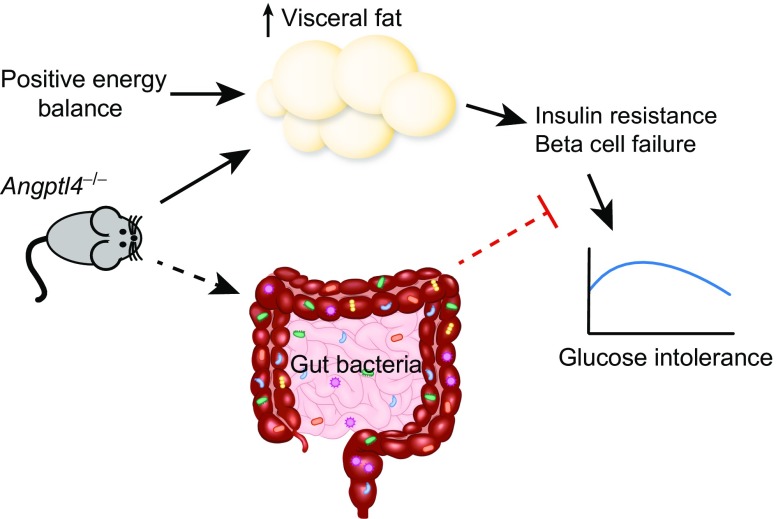
Loss of ANGPTL4 uncouples visceral adiposity from glucose intolerance partly via the gut microbiota. Schematic diagram of the effects of ANGTL4 loss on glucose homeostasis. ANGPTL4 loss raises plasma insulin levels and reduces glucose intolerance via a mechanism that is at least partly dependent on the gut microbiota. Loss of ANGPTL4 also increases visceral adipose tissue mass. Hence, loss of ANGPTL4 uncouples visceral obesity from glucose intolerance. Solid lines, confirmed mechanisms; dashed lines, potential mechanisms

Here we investigated the role of ANGPTL4 in metabolic dysfunction in mice with diet-induced obesity. As shown previously [9, 10, 16, 29], *Angptl4*^−/−^ mice gained more weight, had elevated fat mass, displayed increased adipose LPL activity and LPL protein abundance, and had lower plasma triacylglycerol levels compared with WT mice. Unexpectedly, despite elevated visceral fat mass and inflammation, mice lacking ANGPTL4 had improved glucose tolerance. Interestingly, suppression of the gut bacteria using antibiotics largely abolished the increased glucose tolerance in *Angptl4*^−/−^ mice. Together, our results indicate that loss of ANGPTL4 uncouples visceral obesity from glucose intolerance partly via the gut microbiota (Fig. 6).

In contrast to the well-established function of ANGPTL4 in lipid metabolism, much less is known about its role in glucose metabolism. Previously, we found that mice overexpressing *Angptl4* have impaired glucose tolerance and reduced peripheral insulin sensitivity [15]. Conversely, *Angptl4*^−/−^ mice fed a high (saturated)-fat diet were reported to have improved glucose tolerance [30], although these data were likely confounded by the development of an acute phase response and chylous ascites [16]. Our present data indicate that, despite being heavier, mice lacking ANGPTL4 also have improved glucose tolerance when fed a diet rich in unsaturated fatty acids, in the absence of an acute phase response.

The improved glucose tolerance in *Angptl4*^−/−^ mice was accompanied by elevated insulin levels but not increased insulin sensitivity, suggesting the lower plasma glucose levels are caused by increased insulin secretion. Since lipids can potentiate insulin secretion by pancreatic beta cells [23, 31, 32], we hypothesised that the increased insulin levels in *Angptl4*^−/−^ mice may be due to increased lipid uptake in beta cells as a result of loss of LPL inhibition. Interestingly, LPL does not appear to be localised on the beta cell surface but is primarily within the pancreatic beta cells [33], implying that LPL is not involved in lipid uptake by the beta cell. Furthermore, mice with beta cell-specific overexpression of LPL and increased LPL activity were found to be hyperglycaemic during a glucose tolerance test [24]. Paradoxically, a similar phenotype was observed in beta cell-specific LPL knockout mice. Accordingly, it is difficult to assess the potential role of beta cell LPL in the effect of ANGPTL4 loss on glucose tolerance and insulin secretion. In any case, the effects of ANGPTL4 on glucose tolerance are unlikely to be mediated by beta cell-derived ANGPTL4, as *Angptl4* is minimally expressed in pancreatic islets and beta cells [34]. In agreement with this notion, loss of ANGPTL4 did not significantly affect glucose-stimulated insulin secretion in isolated pancreatic islets. Furthermore, loss of ANGPTL4 did not influence the abundance of pancreatic islets or insulin-staining intensity in the islets.

Interestingly, it was reported that chow-fed *Angptl4*^−/−^ mice have impaired glucose tolerance resulting from impaired insulin secretion and dysmorphic islets [34]. The reason for the discrepancy of this finding with our results remains unclear but could be related to differences in diet. In line with this notion, whole-body *Angptl4*-transgenic mice fed chow or a semi-purified low-fat diet are only marginally glucose intolerant compared with WT mice, yet become highly glucose intolerant on a high-fat diet [14].

Hinting towards a detrimental effect of ANGPTL4 on glucose tolerance in humans, plasma ANGPTL4 levels appear to be positively correlated with fasting blood glucose levels [35]. Intriguingly, human genetic studies suggest a link between LPL/ANGPTL4 and glucose metabolism. Specifically, a gain-of-function variant of *LPL* was associated with higher insulin sensitivity, lower fasting glucose and a lower risk of type 2 diabetes [36]. Conversely, a loss-of-function variant of *LPL* was associated with higher risk of type 2 diabetes [36]. Another study found that gain-of-function alleles of *LPL* and loss-of-function alleles of *ANGPTL4* were associated with lower risk of type 2 diabetes [37]. Whether these effects are specifically connected to LPL in beta cells or elsewhere remains unclear.

Our data suggest that the effects of ANGPTL4 loss on glucose tolerance are partly dependent on the gut microbiota. There is growing evidence that alterations in gut microbiota composition can modulate insulin sensitivity [27, 38–40] and insulin secretion [25, 26]. For example, germ-free and antibiotic-treated mice have improved insulin sensitivity compared with conventionally-raised or untreated mice [38–40]. Furthermore, the difference in insulin secretion between different mouse strains could largely be recapitulated via microbial transfer [25]. In human participants, transfer of faecal microbiota from lean donors to recipients with the metabolic syndrome increased insulin sensitivity [41].

One microbial family that has been shown to be positively correlated with plasma insulin levels is Clostridiaceae [25]. Interestingly, compared with WT mice, we found a higher relative abundance of Clostridiaceae in the colon of *Angptl4*^−/−^ mice, concurrent with elevated plasma insulin levels. However, because certain Clostridiaceae family members also positively correlate with obesity and other metabolic perturbations [42, 43], in our study it is difficult to link Clostridiaceae specifically to increased insulin secretion. Interestingly, in human participants, ingestion of the *Lactobacillus* strain *Lactobacillus reuteri* for 4 weeks improved glucose-induced insulin secretion [26]. In our study, colonic abundance of *Lactobacillus* was almost 2-fold higher in *Angptl4*^−/−^ mice (15.82%) than WT mice (8.82%). It can thus be speculated that an increase in *Lactobacillus* might contribute to elevated insulin levels in *Angptl4*^−/−^ mice.

The gut microbiota may affect glucose metabolism via several mechanisms, including via altered production of SCFAs and LPS. SCFAs may promote insulin secretion via several mechanisms, including via the intestinal G protein-coupled receptor 43 (GPCR43), either dependent [44] or independent [45] of glucagon-like peptide 1 (GLP-1) secretion. In addition, acetate may increase glucose-stimulated insulin secretion via activation of the parasympathetic nervous system [46]. In our study, however, improved glucose tolerance in *Angptl4*^−/−^ mice was associated with lower SCFA levels in the caecum (butyrate) and colon (propionate and butyrate).

Another candidate that may link the gut microbiota to glucose tolerance is LPS. In our study, elevated plasma insulin levels in *Angptl4*^−/−^ mice were associated with higher circulating LPS levels vs WT controls. However, conflicting results exist on the role of LPS in insulin secretion, showing an inhibitory and stimulatory effect [47, 48]. Another study reported that LPS does not affect plasma insulin levels [38], rendering it difficult to link the higher LPS levels in *Angptl4*^−/−^ mice to elevated plasma insulin levels. Hence, SCFA and LPS are probably not involved in the effect of the gut bacteria on insulin levels in *Angptl4*^−/−^ mice.

Interestingly, we found an increased abundance of equol-producing bacteria, including the Coriobacteriaceae family, specifically of the genus *Adlercreutzia*, and Lactobacillus [49]. As equol improves glucose tolerance [50], improved glucose tolerance in *Angptl4*^−/−^ mice might be caused by altered production of equol. However, future studies should investigate the role of equol and other potential candidates outlined in this study in regulating insulin levels and glucose tolerance.

In conclusion, we show that loss of ANGPTL4 in mice with diet-induced obesity promotes visceral obesity while improving glucose tolerance. Suppression of the gut bacteria by antibiotics largely abolished differences in glucose tolerance between WT and *Angptl4*^−/−^ mice, suggesting that ANGPTL4 influences glucose tolerance partly via the gut bacteria. Further studies are warranted to provide additional evidence to support the targeting of ANGPTL4 in the treatment of metabolic disorders.

## Electronic supplementary material

ESM (PDF 734 kb)

## Data Availability

The sequencing data and other raw data are available from the authors upon request.

## Abbreviations

ALT: Alanine aminotransferase
ANGPTL4: Angiopoietin-like 4
LPL: Lipoprotein lipase
LPS: Lipopolysaccharide
rRNA: Ribosomal RNA
SCFA: Short-chain fatty acid
WT: Wild-type

## References

[1] Redinger RN (2007). The pathophysiology of obesity and its clinical manifestations. Gastroenterol Hepatol.

[2] Voshol PJ, Rensen PCN, van Dijk KW (2009). Effect of plasma triglyceride metabolism on lipid storage in adipose tissue: studies using genetically engineered mouse models. Biochim Biophys Acta.

[3] Kersten S (2014). Physiological regulation of lipoprotein lipase. Biochim Biophys Acta.

[4] Davies BSJ, Beigneux AP, Barnes RH (2010). GPIHBP1 is responsible for the entry of lipoprotein lipase into capillaries. Cell Metab.

[5] Beigneux AP, Davies BSJ, Gin P (2007). Glycosylphosphatidylinositol-anchored high-density lipoprotein-binding protein 1 plays a critical role in the lipolytic processing of chylomicrons. Cell Metab.

[6] Dijk W, Kersten S (2016). Regulation of lipid metabolism by angiopoietin-like proteins. Curr Opin Lipidol.

[7] Dijk W, Heine M, Vergnes L (2015). ANGPTL4 mediates shuttling of lipid fuel to brown adipose tissue during sustained cold exposure. elife.

[8] Catoire M, Alex S, Paraskevopulos N (2014). Fatty acid-inducible ANGPTL4 governs lipid metabolic response to exercise. Proc Natl Acad Sci U S A.

[9] Kroupa O, Vorrsjö E, Stienstra R (2012). Linking nutritional regulation of Angptl4, Gpihbp1, and Lmf1 to lipoprotein lipase activity in rodent adipose tissue. BMC Physiol.

[10] Mattijssen F, Alex S, Swarts HJ (2014). Angptl4 serves as an endogenous inhibitor of intestinal lipid digestion. Mol Metab.

[11] Köster A, Chao YB, Mosior M (2005). Transgenic angiopoietin-like (angptl)4 overexpression and targeted disruption of angptl4 and angptl3: regulation of triglyceride metabolism. Endocrinology.

[12] Xu A, Lam MC, Chan KW (2005). Angiopoietin-like protein 4 decreases blood glucose and improves glucose tolerance but induces hyperlipidemia and hepatic steatosis in mice. Proc Natl Acad Sci U S A.

[13] Wang Y, Liu LM, Wei L (2016). Angiopoietin-like protein 4 improves glucose tolerance and insulin resistance but induces liver steatosis in high-fat-diet mice. Mol Med Rep.

[14] Mandard S, Zandbergen F, van Straten E (2006). The fasting-induced adipose factor/angiopoietin-like protein 4 is physically associated with lipoproteins and governs plasma lipid levels and adiposity. J Biol Chem.

[15] Lichtenstein L, Berbée JFP, van Dijk SJ (2007). Angptl4 upregulates cholesterol synthesis in liver via inhibition of LPL- and HL-dependent hepatic cholesterol uptake. Arterioscler Thromb Vasc Biol.

[16] Lichtenstein L, Mattijssen F, de Wit NJ (2010). Angptl4 protects against severe proinflammatory effects of saturated fat by inhibiting fatty acid uptake into mesenteric lymph node macrophages. Cell Metab.

[17] Oteng A-B, Bhattacharya A, Brodesser S (2017). Feeding *Angptl4*^−/−^ mice *trans* fat promotes foam cell formation in mesenteric lymph nodes without leading to ascites. J Lipid Res.

[18] Janssen AWF, Houben T, Katiraei S (2017). Modulation of the gut microbiota impacts non-alcoholic fatty liver disease: a potential role for bile acids. J Lipid Res.

[19] Basciano H, Federico L, Adeli K (2005). Fructose, insulin resistance, and metabolic dyslipidemia. Nutr Metab.

[20] Chung S, Parks JS (2015). Dietary cholesterol effects on adipose tissue inflammation. Curr Opin Lipidol.

[21] Janssen AWF, Dijk W, Boekhorst J (2017). ANGPTL4 promotes bile acid absorption during taurocholic acid supplementation via a mechanism dependent on the gut microbiota. Biochim Biophys Acta Mol Cell Biol Lipids.

[22] Ijssennagger N, Belzer C, Hooiveld GJ (2015). Gut microbiota facilitates dietary heme-induced epithelial hyperproliferation by opening the mucus barrier in colon. Proc Natl Acad Sci.

[23] Cruz WS, Kwon G, Marshall CA (2001). Glucose and insulin stimulate heparin-releasable lipoprotein lipase activity in mouse islets and INS-1 cells. A potential link between insulin resistance and beta-cell dysfunction. J Biol Chem.

[24] Pappan KL, Pan Z, Kwon G (2005). Pancreatic β-cell lipoprotein lipase independently regulates islet glucose metabolism and normal insulin secretion. J Biol Chem.

[25] Kreznar JH, Keller MP, Traeger LL (2017). Host genotype and gut microbiome modulate insulin secretion and diet-induced metabolic phenotypes. Cell Rep.

[26] Simon MC, Strassburger K, Nowotny B (2015). Intake of *Lactobacillus reuteri* improves incretin and insulin secretion in glucose-tolerant humans: a proof of concept. Diabetes Care.

[27] Bäckhed F, Ding H, Wang T (2004). The gut microbiota as an environmental factor that regulates fat storage. Proc Natl Acad Sci U S A.

[28] Bäckhed F, Manchester JK, Semenkovich CF, Gordon JI (2007). Mechanisms underlying the resistance to diet-induced obesity in germ-free mice. Proc Natl Acad Sci U S A.

[29] Sukonina V, Lookene A, Olivecrona T, Olivecrona G (2006). Angiopoietin-like protein 4 converts lipoprotein lipase to inactive monomers and modulates lipase activity in adipose tissue. Proc Natl Acad Sci U S A.

[30] Lee E-C, Landes GM, Chung K, et al; Lexicon Pharmaceuticals, Inc, Monoclonal antibodies against ANGPTL4. US Patent 2006/0222645 A1. 6 Jan 2006

[31] Mulder H, Yang S, So M (2004). Inhibition of lipase activity and lipolysis in rat islets reduces insulin secretion. Diabetes.

[32] Koyama K, Chen G, Wang MY (1997). Beta-cell function in normal rats made chronically hyperleptinemic by adenovirus-leptin gene therapy. Diabetes.

[33] Nyrén R, Chang CL, Lindström P (2012). Localization of lipoprotein lipase and GPIHBP1 in mouse pancreas: effects of diet and leptin deficiency. BMC Physiol.

[34] Kim H-K, Kwon O, Park K-H (2017). Angiopoietin-like peptide 4 regulates insulin secretion and islet morphology. Biochem Biophys Res Commun.

[35] Mehta N, Qamar A, Qu L (2014). Differential association of plasma angiopoietin-like proteins 3 and 4 with lipid and metabolic traits. Arterioscler Thromb Vasc Biol.

[36] Lotta LA, Gulati P, Day FR (2017). Integrative genomic analysis implicates limited peripheral adipose storage capacity in the pathogenesis of human insulin resistance. Nat Genet.

[37] Liu DJ, Peloso GM, Yu H (2017). Exome-wide association study of plasma lipids in >300,000 individuals. Nat Genet.

[38] Caesar R, Reigstad CS, Bäckhed HK (2012). Gut-derived lipopolysaccharide augments adipose macrophage accumulation but is not essential for impaired glucose or insulin tolerance in mice. Gut.

[39] Rabot S, Membrez M, Bruneau A (2010). Germ-free C57BL/6J mice are resistant to high-fat-diet-induced insulin resistance and have altered cholesterol metabolism. FASEB J.

[40] Hwang I, Park YJ, Kim YR (2015). Alteration of gut microbiota by vancomycin and bacitracin improves insulin resistance via glucagon-like peptide 1 in diet-induced obesity. FASEB J.

[41] Vrieze A, Van Nood E, Holleman F (2012). Transfer of intestinal microbiota from lean donors increases insulin sensitivity in individuals with metabolic syndrome. Gastroenterology.

[42] Ussar S, Griffin NW, Bezy O (2015). Interactions between gut microbiota, host genetics and diet modulate the predisposition to obesity and metabolic syndrome. Cell Metab.

[43] Karlsson FH, Tremaroli V, Nookaew I (2013). Gut metagenome in European women with normal, impaired and diabetic glucose control. Nature.

[44] Priyadarshini M, Wicksteed B, Schiltz GE (2016). SCFA receptors in pancreatic β cells: novel diabetes targets?. Trends Endocrinol Metab.

[45] Priyadarshini M, Villa SR, Fuller M (2015). An acetate-specific GPCR, FFAR2, regulates insulin secretion. Mol Endocrinol.

[46] Perry RJ, Peng L, Barry NA (2016). Acetate mediates a microbiome–brain–β-cell axis to promote metabolic syndrome. Nature.

[47] Amyot J, Semache M, Ferdaoussi M (2012). Lipopolysaccharides impair insulin gene expression in isolated islets of langerhans via toll-like receptor-4 and NF-kB signalling. PLoS One.

[48] Nguyen AT, Mandard S, Dray C (2014). Lipopolysaccharides-mediated increase in glucose-stimulated insulin secretion: involvement of the GLP-1 pathway. Diabetes.

[49] Setchell KDR, Clerici C (2010). Equol: history, chemistry, and formation. J Nutr.

[50] Cheong SH, Furuhashi K, Ito K (2014). Antihyperglycemic effect of equol, a daidzein derivative, in cultured L6 myocytes and ob/ob mice. Mol Nutr Food Res.

