# Supplementary material for: Loss of angiopoietin-like 4 (ANGPTL4) in mice with diet-induced obesity uncouples visceral obesity from glucose intolerance partly via the gut microbiota
Source: Diabetologia. 2018 Mar 3;61(6):1447–58. doi: 10.1007/s00125-018-4583-5 (PMC6449003; doi:10.1007/s00125-018-4583-5)
Supplement: Supplementary file 1 — (PDF 734 kb) [file 125_2018_4583_MOESM1_ESM.pdf]

Electronic Supplementary Material for:

Loss of angiopoietin-like 4 (ANGPTL4) in mice with diet-induced obesity uncouples visceral obesity from glucose intolerance partly via the gut microbiota

Aafke W.F. Janssen[1], Saeed Katiraei[2,3], Barbara Bartosinska[4,5,6], Daniel Eberhard[4,5,6], Ko Willems van Dijk[2,3,7], Sander Kersten[1]

[1]Nutrition, Metabolism and Genomics Group, Division of Human Nutrition and Health, Wageningen University, Stippeneng 4, 6708 WE Wageningen, the Netherlands

[2]Department of Human Genetics, Leiden University Medical Center, Leiden, the Netherlands

[3]Einthoven Laboratory for Experimental Vascular Medicine, Leiden University Medical Center, Leiden, the Netherlands

[4]Institute of Metabolic Physiology, Department of Biology, Heinrich Heine University Düsseldorf, Düsseldorf, Germany

[5]Institute for Beta Cell Biology, German Diabetes Center, Leibniz Center for Diabetes Research, Heinrich Heine University Düsseldorf, Düsseldorf, Germany

[6]German Center for Diabetes Research (DZD) München Neuherberg, Germany

[7]Division of Endocrinology, Department of Medicine, Leiden University Medical Center, Leiden, the Netherlands

Correspondence should be addressed to: Sander Kersten, PhD, Nutrition, Metabolism and Genomics group, Division of Human Nutrition and Health, Wageningen University, Stippeneng 4, 6708 WE Wageningen, The Netherlands. Phone: +31 317 485787; Email: sander.kersten@wur.nl.

ESM Methods

Intraperitoneal glucose tolerance test

Mice were placed in clean cages without food and fructose water was replaced by tap watehr with or without the antibiotic mixture at 8.00h. After 5 hours of fasting, mice were weighed and a blood sample was drawn. Mice were injected intraperitoneally with glucose (0.8 g/kg bodyweight) (Baxter, Utrecht, the Netherlands) and blood was drawn at specific time points for measurement of glucose using Accu-chek Compact (study 1) (Roche Diagnostics, Almere, the Netherlands) or Glucofix Tech (study 2) (Menarini, Valkenswaard, the Netherlands).

In study 1, plasma concentrations of insulin were measured after 5 hours of fasting and in study 2 after 5 hours of fasting and 20 and 60 minutes after glucose injection. The insulin measurement was done according to manufacturer's instructions (Crystal Chem, Downers Grove, USA).

Intraperitoneal insulin tolerance test

Mice were fasted for 5 hours prior to the insulin tolerance test. During this period fructose water was replaced by tap water with or without the antibiotic mixture. Blood samples were collected from the tail vein immediately before (t=0 min) and at selected time points after intraperitoneal insulin injection (0.75U/kg bodyweight)(Actrapid, Novo Nordisk, Alphen aan de Rijn, the Netherlands). Glucose was measured using Glucofix Tech.

RNA isolation and qPCR

Total RNA was extracted using TRIzol reagent (Life technologies, Bleiswijk, Netherlands). RNA from mesenteric fat depots and pancreatic islets was purified using the RNeasy minikit (Qiagen, Venlo, The Netherlands). RNA was reverse transcribed to cDNA using the iScript cDNA synthesis kit (Bio-Rad Laboratories, Veenendaal, the Netherlands). Changes in gene expression were determined by real-time PCR on a CFX384 Real-Time PCR detection system (Bio-Rad) by using SensiMix (Bioline, GC biotech, Alphen aan den Rijn, the Netherlands). The data were analyzed using the Bio-Rad CFX Manager 3.0. The housekeeping genes *36b4* was used for normalization. Sequences of the primers used are listed below.

	Primer Sequence	
Name	Forward	Reverse
36b4	ATGGGTACAAGCGCGTCCTG	GCCTTGACCTTTTCAGTAAG
Cd68	CCAATTCAGGGTGGAAGAAA	CTCGGGCTCTGATGTAGGTC
F4/80 (Emr1)	CTTTGGCTATGGGCTTCCAGTC	GCAAGGAGGACAGAGTTTATCGTG
Cxcl2 (MCP1)	CCCAATGAGTAGGCTGGAGA	TCTGGACCCATTCCTTCTTG
Il6	CTTCCATCCAGTTGCCTTCTTG	AATTAAGCCTCCGACTTGTGAAG
Il1ra	AAATCTGCTGGGGACCCTAC	TGAGCTGGTTGTTTCTCAGG
Lpl	CAGCTGGGCCTAACTTTGAG	GACCCCCTGGTAAATGTGTG
Angptl4	GTTTGCAGACTCAGCTCAAGG	CCAAGAGGTCTATCTGGCTCTG

Mouse pancreatic islet isolation

Pancreatic islets were isolated from wild-type and *Angptl4*-/- mice using Liberase TL Research Grade (Roche) as previously described [1]. Briefly, Liberase TL was dissolved in low glucose DMEM (Gibco) and 2-3 mL was injected into the bile duct. After incubation of the perfused pancreas at 37°C, the digestion was stopped by adding DMEM containing FCS. The islet suspension was subsequently filtered (420 µm mesh) and centrifuged using Histopaque 1077 (Sigma-Aldrich). Islets were collected from the interphase between Histopaque 1077 and DMEM, and washed twice with CMRL medium (Gibco) containing 15% heat-inactivated FCS, 11 mM glucose, 0.15% $NaHCO_3$, 50 µM β-mercaptoethanol and 1% penicillin/streptomycin. Subsequently, pancreatic islets were collected for RNA isolation.

Glucose-stimulated insulin secretion

To study the glucose-stimulated insulin release of islets of wild-type and *Angptl4*-/- mice, groups of 8 islets were fasted for 1 hour in Krebs Ringer HEPES buffer (KRH, 15 mM HEPES, 5 mM KCl, 120 mM NaCl, 2 mM CaCl2, 0.01 mM glycine, 24 mM NaHCO3 and 1 mg/ml bovine serum albumin) containing 2 mM glucose. Thereafter, the islets were sequentially stimulated for 1 hour in KRH buffer supplemented with 2 mM and 20 mM glucose and subsequently lysed using radioimmuno-precipitation assay (RIPA) buffer (50 mM Tris-HCl (pH 7.4), 150 mM NaCl, 1 mM EDTA, 0.25% Na-deoxycholate, 1% IGEPAL, 1mM sodium orthovanadate, 1mM sodium fluoride).

Secreted insulin as well as insulin content was quantified by ELISA (ultra-sensitive rat insulin ELISA, Crystal Chem) in combination with an Infinite M200 NanoQuant microplate reader (Tecan, Männedorf, Switzerland). For quantification, secreted insulin measured in supernatants was normalized to the protein content in cell lysate and expressed as percentage of the mean of the wild-type at 2 mM glucose.

Western blot

Mesenteric fat pads were lysed in ice-cold Pierce IP lysis buffer (Thermo Fisher Scientific, Breda, the Netherlands) containing protease and phosphatase inhibitors (Roche). Lysates were centrifuged 4 times for 10min at 13000g to remove fat droplets. 15 µg of protein was loaded on a Criterion TGX gel, 8-16% and subsequently transferred onto a PVDF membrane using a Transblot Turbo system (Bio-Rad). After blocking in TBS, 0.1% Tween-20 and 5% nonfat dry milk, the membranes were incubated overnight with a goat anti-mouse LPL antibody (gift of Dr. André Bensadoun, Cornell University, Ithaca, NY, USA) [2] or a rabbit anti-goat HSP90 (Cell Signalling, #4874) antibody, both at 1:2000 dilutions. Finally, the blots were exposed to Enhanced Chemiluminescent substrate (Bio-Rad) for the visualization of the protein bands by using the ChemiDoc MP Imaging System (Bio-Rad).

DNA extraction

In study 1, fecal samples derived from the second part of the colon were suspended in 10 mM Tris, 1mM EDTA, 0.5% SDS and 0.2 mg/mL Proteinase K (Thermo Fisher Scientific, Breda, the Netherlands). After addition of 0.1-0.25 mm and 4 mm glass beads, buffered phenol (Invitrogen, Carlsbad, USA) was added and cells were lysed by mechanical disruption using a bead beater (MP biomedicals, Santa Ana, USA) for 3 minutes. DNA was subsequently extracted

using phenol:chloroform:isoamylalcohol [25:24:1] (Invitrogen, Carlsbad, USA), precipitated with isopropanol and washed with 70% ethanol.
In study 2, fecal samples (~15-60 mg) derived from the second part of the colon were suspended in 500 µL S.T.A.R. buffer (Roche). After addition of 0.1 mm zirconia and 2.5 mm glass beads (BioSpec, Bartlesville, USA), cells were lysed by mechanical disruption using a bead beater (MP biomedicals, Santa Ana, USA) for 3x1 minute. DNA was subsequently extracted and purified using Maxwell 16 System (Promega). In brief, homogenates (250 µL) were transferred to a prefilled reagent cartridge (Maxwell® 16 Tissue LEV Total RNA Purification Kit, Custom-made, Promega). Sixteen samples were processed at the same time. After 30 minutes the purification process was completed and DNA was eluted in 50 µL of water (Nuclease free)(Promega).

16S rRNA gene sequencing
For 16S rRNA gene sequencing DNA samples were send to the Broad Institute of MIT and Harvard (Cambridge, USA). Microbial 16S rRNA gene was amplified targeting the hyper-variable region V4 using forward primer 515F (5'-GTGCCAGCMGCCGCGGTAA-3') and the reverse primer 806R (5'- GGACTACHVGGGTWTCTAAT-3'). The cycling conditions consisted of an initial denaturation of 94°C for 3 min, followed by 25 cycles of denaturation at 94°C for 45 sec, annealing at 50 °C for 60 sec, extension at 72°C for 5 min, and a final extension at 72°C for 10 min. Sequencing was performed using the Illumina MiSeq platform (San Diego, CA, USA) generating paired-end reads of 175 bp in length in each direction. Overlapping paired-end reads were subsequently aligned. Detailed of this protocol are as previously described [3].
Raw sequence data quality was assessed using FastQC, version: 0.11.2 (http://www.bioinformatics.babraham.ac.uk/projects/fastqc/). Reads quality was checked with Sickle, version: 1.33 (https://github.com/najoshi/sickle) and low quality reads were removed. For visualising the taxonomic composition of the fecal microbiota and further beta diversity analysis, QIIME, version: 1.9.0 was used [4]. In brief, closed reference OTU picking with 97% sequence similarity against GreenGenes 13.8 reference database was done. Jackknifed beta-diversity of unweighted UniFrac distances with 10 jackknife replicates was measured at rarefaction depth of 22000 reads/sample. For statistical significance, biological relevance and visualisation we used linear discriminant analysis (LDA) effect size (LEfSe) method (https://bitbucket.org/biobakery/biobakery/wiki/lefse)[5]. The sequencing data are available from the authors upon request.

Bacterial 16S rRNA gene quantification
Standard curves were constructed by amplifying 16S rRNA gene using fecal DNA of the control group using the universal 16S primers 27F (5'-GTTTGATCCTGGCTCAG-3') and 1492R (5'-CGGCTACCTTGTTACGAC-3'). The cycling conditions consisted of an initial denaturation of 95°C for 5 min, followed by 35 cycles of denaturation at 95°C for 30 sec, annealing at 52°C for 40 sec and extension at 72°C for 90 sec and a final extension at 72°C for 7 min. Amplicon size was verified by agarose gel electrophoresis and amplicon was purified using a commercial available PCR purification kit (Thermo Fisher Scientific, Breda, the Netherlands). After the

DNA concentration was quantitated and copy number was calculated, a serial dilution ranging from 10^8 to 10^1 16S rRNA gene copies/µL was generated.

Real-time PCR for the 16S rRNA gene was performed using fecal DNA samples from study 2 and standards on a CFX384 Real-Time PCR detection system (Bio-Rad Laboratories, Veenendaal, Netherlands) with SensiMix (Bioline, GC biotech, Alphen aan den Rijn, Netherlands). 16S rRNA gene was amplified using the forward primer 1369F (5'-CGGTGAATACGTTCYCGG-3')[6] and the reverse primer 1492R (5'-GGWTACCTTGTTACGACTT-3')[7]. The cycling conditions consisted of an initial denaturation of 95°C for 5 min, followed by 40 cycles of denaturation at 95°C for 15 sec, annealing at 60 °C for 30 sec and extension at 72°C for 30 sec. The data were analyzed using the Bio-Rad CFX Manager 3.0.

References

1. Yesil P, Michel M, Chwalek K, et al (2009) A new collagenase blend increases the number of islets isolated from mouse pancreas. Islets 1:185–190.
2. Weinstein MM, Yin L, Beigneux AP, et al (2008) Abnormal patterns of lipoprotein lipase release into the plasma in GPIHBP1-deficient mice. J Biol Chem 283:34511–34518.
3 Gevers D, Kugathasan S, Denson LA, et al (2014) The treatment-naive microbiome in new-onset Crohn's disease. Cell Host Microbe 15:382–392.
4. Caporaso JG, Kuczynski J, Stombaugh J, et al (2010) QIIME allows analysis of high-throughput community sequencing data. Nat Methods 7:335–336.
5. Segata N, Izard J, Waldron L, et al (2011) Metagenomic biomarker discovery and explanation. Genome Biol 12:R60.
6. Suzuki MT, Taylor LT, DeLong EF (2000) Quantitative analysis of small-subunit rRNA genes in mixed microbial populations via 5'-nuclease assays. Appl Environ Microbiol 66:4605–4614.
7. Weisburg WG, Barns SM, Pelletier DA, Lane DJ (1991) 16S ribosomal DNA amplification for phylogenetic study. J Bacteriol 173:697–703.

ESM Table 1. Relative abundance of microbiota in colonic luminal content

Phylum	Class	Order	Family	Genus	Species	Wild-type (%)	*Angptl4* $^{-/-}$ *(%)*	FC	P-value*
Actinobacteria						0.67	1.68	2.5	0.004
	Coriobacteriia					0.67	1.68	2.5	0.004
		Coriobacteriales				0.67	1.68	2.5	0.004
			Coriobacteriaceae			0.67	1.68	2.5	0.004
				Adlercreutzia		0.67	1.68	2.5	0.004
Bacteroidetes						4.61	5.86	1.3	ns
	Bacteroidia					4.61	5.86	1.3	ns
		Bacteriodales				4.61	5.86	1.3	ns
			Unidentified			2.04	2.46	1.2	ns
			Bacteroidaceae			0.95	1.14	1.2	ns
				Bacteroides		0.95	1.14	1.2	ns
			S24-7			1.61	2.26	1.4	ns
				Unidentified		1.61	2.26	1.4	ns
Deferribacteres						4.67	4.26	-1.1	ns
	Deferiibacteres					4.67	4.26	-1.1	ns
		Deferribacterales				4.67	4.26	-1.1	ns
			Deferribacteraceae			4.67	4.26	-1.1	ns
				Mucispirillum		4.67	4.26	-1.1	ns
					Mucispirillum schaedleri	4.67	4.26	-1.1	ns
Firmicutes						84.95	82.24	-1.0	ns
	Bacilli					8.82	15.82	1.8	0.023
		Lactobacillales				8.82	15.82	1.8	0.023
			Lactobacillaceae			8.82	15.82	1.8	0.023
				Lactobacillus		8.82	15.82	1.8	0.023
	Clostridia					29.88	46.49	1.6	0.011
		Clostridiales				29.88	46.49	1.6	0.011
			Unidentified			19.04	23.21	1.2	ns
			Clostridiaceae			3.84	14.19	3.7	0.001
				Unidentified		0.00	1.97	>2000	<0,001
				SMB53		3.84	12.22	3.2	0.001
			Lachnospiraceae			2.22	1.61	-1.4	ns
				Unidentified		2.22	1.61	-1.4	ns
			Ruminococcaceae			4.78	7.49	1.6	ns
				Unidentified		0.90	3.67	4.1	ns
				Oscillospira		3.23	3.15	1.0	ns
				Ruminococcus		0.64	0.66	1.0	ns
	Erysipelotrichi					46.25	19.93	-2.3	0.016
		Erysipelotricales				46.25	19.93	-2.3	0.016
			Erysipelotrichaceae			46.25	19.93	-2.3	0.016
				Allobaculum		46.25	19.93	-2.3	0.016
Proteobacteria						5.09	5.96	1.2	ns
	Deltaproteobacteria					5.09	5.96	1.2	ns
		Desulfovibrionales				5.09	5.96	1.2	ns
			Desulfovibrionaceae			5.09	5.96	1.2	ns
				Bilophila		2.84	4.09	1.4	ns
				Desulfovibrio		2.26	1.87	-1.2	ns

Abundance treshold for presentation in the table >0.5% in at least one of the two genotypes
* significance according to unpaired Wilcoxon rank-sum test
